# Self-Powered WSN for Distributed Data Center Monitoring

**DOI:** 10.3390/s16010057

**Published:** 2016-01-02

**Authors:** Davide Brunelli, Roberto Passerone, Luca Rizzon, Maurizio Rossi, Davide Sartori

**Affiliations:** 1 Department of Industrial Engineering (DII), University of Trento, via Sommarive 9, Povo (TN) I-38123, Italy; davide.brunelli@unitn.it (D.B.); maurizio.rossi@unitn.it (M.R.); davide.sartori.1@unitn.it (D.S.); 2 Department of Information Engineering and Computer Science (DISI), University of Trento, via Sommarive 9, Povo (TN) I-38123, Italy; roberto.passerone@unitn.it

**Keywords:** TEGs, energy harvesting, batteryless WSN

## Abstract

Monitoring environmental parameters in data centers is gathering nowadays increasing attention from industry, due to the need of high energy efficiency of cloud services. We present the design and the characterization of an energy neutral embedded wireless system, prototyped to monitor *perpetually* environmental parameters in servers and racks. It is powered by an energy harvesting module based on Thermoelectric Generators, which converts the heat dissipation from the servers. Starting from the empirical characterization of the energy harvester, we present a power conditioning circuit optimized for the specific application. The whole system has been enhanced with several sensors. An ultra-low-power micro-controller stacked over the energy harvesting provides an efficient power management. Performance have been assessed and compared with the analytical model for validation.

## 1. Introduction

Nowadays, IT systems are more distributed than ever, Data Centers are spread all over the world providing services for infrastructure virtualization, cloud and IoT applications. Moreover, in the near future, all the small to medium internally company-owned IT infrastructure will be replaced by, so-called, “mega-data centers”, since outsourcing these facilities is much more cost-effective rather than maintaining an own infrastructure and hiring IT managers [1]. In this scenario, the need for security, reliability and the maximization of service up-time can be achieved only relying on automatic incident remediation solutions, both on the cyber-security and on the physical side.

Another important challenge for service providers is to adapt their infrastructure to a more environmentally friendly and fossil fuel free economy [2], following and complying with regulations that are currently being issued by governments all over the world. In this regard, all the biggest companies providing cloud services in the IT market [3] (Amazon [4], Google [5], Apple [6], Microsoft [7], Rackspace [8], and IBM [9] for instance) have already introduced renewable energy sources in their supply chain.

Clearly, future “green” data centers will require a completely different design also on the Data Center Infrastructure Management (DCIM) side, to achieve capillary monitoring of the thermal environment and the energy resources, as well as to guarantee security. These features are best achieved using approaches which are as neutral as possible relative to the energy impact, *i.e.*, which work transparently by taking advantage of freely available power from the environment, without using additional energy from batteries or from the wired infrastructure. In fact, one of the most used metrics for evaluating the energy efficiency of a data center, Power Usage Effectiveness (PUE), is defined as the ratio between the total energy used to supply the data center, and the energy used for computation alone. The lower the PUE, the higher the efficiency. The additional energy used from the monitoring infrastructure increases the amount of total energy spent, thus the PUE increases, resulting in a worse utilization of the provided electrical power. The aim of this research is to provide a solution that is beyond the industrial standard and to push forward the state-of-art in terms of energy neutral networked portable electronic devices for monitoring tasks.

We present a batteryless embedded system that has been designed to realize a maintenance-free wireless sensor monitoring infrastructure for data centers. By exploiting wasted heat energy inside data center facilities, the most abundant kind of wasted energy, our monitoring system can provide *continuous* sampling of several environmental parameters—like temperature, humidity and light—and *enhanced security*, thanks to the use of various sensors. Monitoring system already available in the market usually are powered from the grid, burdening the data center PUE. In fact, the energy spent by a monitoring system increases the power consumption of the infrastructure, and consequently worsens the datacenter energy efficiency. Moreover, their sampling rate is lower compared to the proposed system. The Data Center Genome Project, a monitoring system developed by Microsoft Research, samples the environment every 30 s [10]. The sampling period of the solution proposed by Dell, called OpenManage Power Center Temperature Monitoring, can be set to 1, 3, or 6 min [11]. Given the number of nodes that must be installed to instantiate the monitoring network, it would be impracticable to use wires for power supply and for communication. A connector to interface the proposed monitoring device with the data center motherboard does not exist, therefore, new dedicated wires must be placed to deploy a wired sensor network. Using batteries poses the issue of replacing them when exhausted. For these reasons, we explored the utilization of renewable energy sources, and we adopted a wireless solution for our monitoring infrastructure.

The system we propose introduces significant improvements, because it is totally powered by free energy it scavenges from wasted energy, and it monitors the environmental parameters with a shorter sampling period. Moreover, thanks to the smart sinks reduced size, a large number of them can be placed inside a server room, therefore it is possible to perform distributed monitoring of the environment, and to provide a wider cumulative data rate. In case of overheating, the proposed batteryless monitoring device scavenges enough power to operate with less than one second data rate.

We designed, built, and tested a Thermoelectric Generator (TEG) based energy transformer with dedicated conditioning circuitry able to guarantee never-ending supply to the wireless sensing node with as little as 10 ${}^{{}^{\circ}}$C temperature difference across the harvesting stage. Each CPU in the data-center can be equipped with such a device, setting up a complete wireless sensor network able to track the environmental parameters. Our results demonstrate that the batteryless system can operate continuously, regardless of the data-center load, which is the most relevant achievement with respect to the state-of-art in this field.

The article is organized as follows. Section 2 presents the state of the art in terms of energy harvesting for TEGs, and environmental Wireless Sensor Network (WSN). Section 3 describes the design and characterization phase of the hardware unit for energy harvesting and conditioning; Section 4 presents the prototype implementation while, in Section 5, we summarize the results and performance in terms of monitoring reliability and self-sustainability. Finally, Section 6 concludes the work.

## 2. Related Work

According to analysts forecast, the thermoelectric-generation market will increase from $40 millions in 2014 to $950 millions in 2024 [12]. This trend is mainly driven by the automotive sector [13], where the thermal energy generated by combustion engines is so high that by using integrated TEGs it is possible to scavenge hundreds of Watts, and to reduce fuel consumption and carbon emissions at the same time.

However, thermoelectric scavengers can be useful in any scenario where thermal energy is lost in large amounts, such as power electronics and high performance computing. Here, the energy harvested from otherwise wasted heat can be reused for boosting standard cooling systems and/or supply related tasks. In the IT sector, for instance, part of the energy spent in computing is wasted through heat, and major efforts are made to keep the equipment at a safe temperature. Wireless sensors powered by the dissipated heat can lower the system cost by boosting the heat extraction and providing a monitoring infrastructure to enforce the existing DCIM systems. By eliminating power wires, the installation costs decrease, moreover, not having to periodically replace their batteries allows the system to be installed in yet unexplored environments.

The integration of Thermoelectric Coolers (TECs) and TEGs on the CPU’s cooling devices to convert wasted heat into electricity and boost the heat exchange at the same time is an active research field [14,15] but current solutions are usually large and bulky. The amount of scavenged energy is proportional to the heat generated by the CPU that is directly dependent on the kind of task the processor is running, its duration, and the environmental temperature [16]. The recovered energy is small compared to the energy consumed by the CPU itself, therefore it is not enough to supply components of the device it scavenges from, nor to recharge the system’s batteries [17,18]. However, it can be used to provide active cooling to the processor, so as to increase the PUE of the system reducing the energy used for cooling (or, conversely, allows the system to run at a higher clock frequency without causing overheating) [19,20].

Moreover, the rapid growth of networked smart sensors and actuators is about to hit the data center domain, promising the control of environmental parameters that are crucial for the system monitoring. Very low powered wireless sensor nodes can be deployed in the data centers facilities, and run thanks solely to the energy converted from the excess of heat [21]. Providing monitoring with free energy means having a security infrastructure which works in harmony with the data centers without increasing the energy spent, requiring no maintenance, and no additional wiring. Given the needs of the data center operators to collect environmental data to monitor safety in their infrastructure [22], and considering the fact that the WSNs are very well suited for this purpose [23]—since they consume a very limited amount of power—we focused on the development of a wireless monitoring device that can be powered with the energy that the CPUs waste through the heat.

## 3. Thermoelectric Harvesting

The system is used to monitor the health of the host machine. It exploits a thermoelectric generator to convert the thermal gradient of the heat, dissipated from a high performance processor (*host*), into electrical energy to power the smart sensors. The TEG represents the basic component for the conversion of wasted heat into electrical power. It consists of a number of semiconductor elements (p-n junctions) that are thermally connected in parallel (but electrically in series), and enclosed in a package designed to spread the heat on the whole surface (can be metallic or ceramic). Each of these elements exhibits an electromotive force when exposed to a thermal difference (or thermal gradient) due to the Seebeck effect. Since these elements are electrically connected in series, each contribution sums up to build a voltage at the output metal connectors of the device. The generated voltage is proportional to the thermal difference at which the device is exposed to.

**Figure 1:**
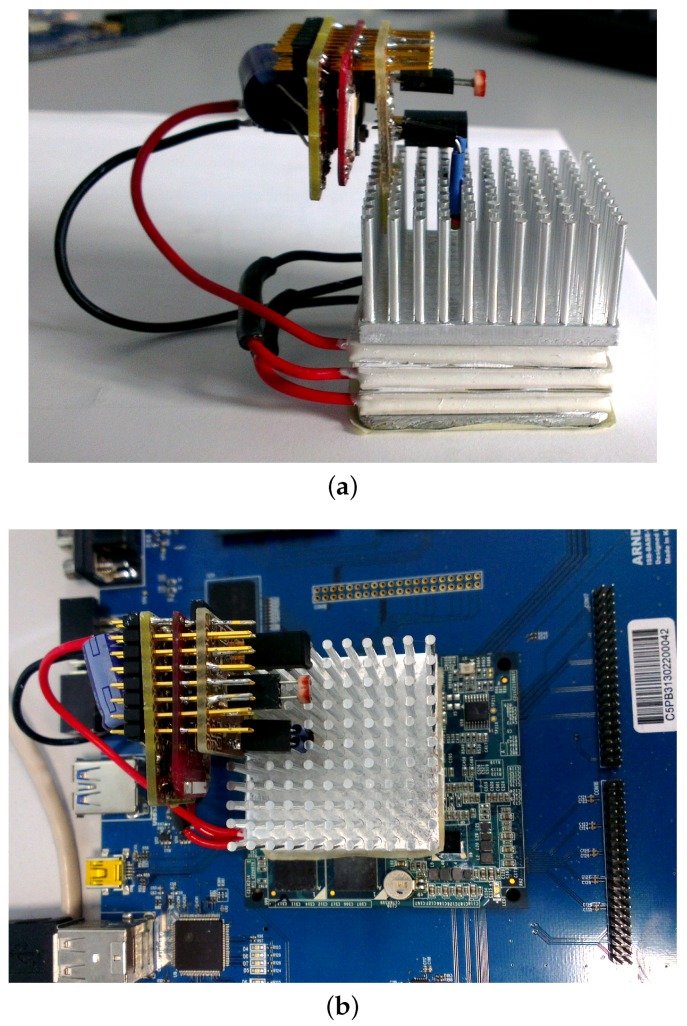
Pictures of the system prototype. (**a**) Side view of the TEG with heat sink, sensors, and transceiver board; (**b**) Top view of the system prototype mounted on top of the target CPU board.

The prototype harvester, consisting of three stacked TEGs, and an electronic circuit for the conditioning and storage of the harvested energy is depicted in Figure 1a. The harvester module is placed on top of the host CPU, and is completed by an aluminum heat sink, as in Figure 1b. The heat sink is needed to keep the heat flowing thought the TEG and ease the thermal exchange (of the excess heat not converted into electricity) with the environment. Removing the heat from the cold side of the TEGs is essential to ensure a continuous electric current from the generator. If not removed, the heat would be retained in the TEG until a thermal equilibrium which stops the generation of electrical power. Each wireless node consists of the thermoelectric harvester, a circuit board for the conditioning circuit and the storage capacitance, a circuit board that houses the sensors, and another board for the microcontroller and the radio chip. Distributed monitoring is therefore achieved by instrumenting the datacenter with hundreds of nodes, one for each CPU or heat generating device. The overall dimension of the node is 40×40×50 mm.

### 3.1. Conditioning Hardware

The electrical power generated by TEGs is generally lower than the supply required by the wireless sensor nodes. Typically, it stands in the order of hundreds of microwatts with voltages in the hundred of millivolts. Therefore, an efficient conditioning circuit is required to raise the generated voltage up to an acceptable level to feed the system, and to accumulate energy. The sensing node operates sporadically in short bursts with a given duty cycle, therefore, the conditioning circuit must contain a storage element to accumulate the energy, and to provide energy on demand. The sizing of the storage elements depends on the kind of application implemented on the wireless node, and on the expected system lifetime.

Operating under a low duty cycle, the monitoring system consumes, on average, a low amount of power, and the application can be supported using a small energy buffer (a capacitance of few millifarads). On the other side, a larger storage unit guarantees the node operations for longer time, even in case there is no power at its input. In any case, the size of the storage elements is driven by the application requirements, because it depends on the amount of time necessary to accumulate the required energy.

**Figure 2:**
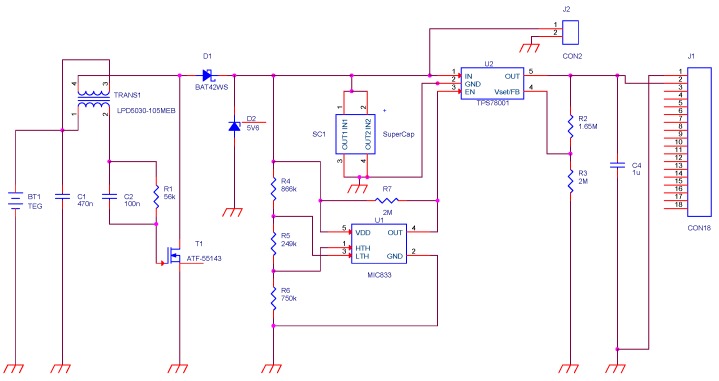
Electronic diagram of the conditioning hardware circuit that converts and stores the energy generated by the TEG, and supplies the WSN.

The complete conditioning circuit, shown in Figure 2, is composed of a cascade of several components, namely: a step-up (boost) converter, a rectifier, a storage unit, a voltage comparator, and a LDO voltage regulator. The initial stage consists of a resonant step-up oscillator that converts a very low-power input voltage (hundreds of millivolts) into a higher voltage output. The resonator circuit multiplicative factor (*σ*) depends on the voltage at its input. The resonator requires at least 220 mV input voltage to let the power through the output with the amount of energy required to charge the storage unit. With an input larger than 290 mV the step-up converter provides a 5 V output voltage, with σ≥15.

Then, the signal is filtered, decoupled, and rectified to be used by the subsequent stage: the storage unit *SC1* which consists of a capacitive element. The storage unit may be made with supercapacitors or solid state batteries depending on the robustness required by the application. For example, with less-frequent, but energy-demanding tasks (e.g., wireless communication) a supercapacitor of a few Farads represents a good solution, since it takes a few hours to charge, with high energy density to supply powerful embedded platforms [16]. We selected a single capacitor aiming to boost recharge time rather than energy density. Section 4.2 describes in details the design choices made with respect to the harvester performance, and the application requirements.

The Low DropOut voltage regulator (LDO) (*U2*) maintains a steady output voltage which is lower than the input voltage source. It is needed to supply the node with a constant, proper voltage even when the voltage stored in the supercapacitor is higher, so to limit the current drop. The LDO is controlled by a comparator (*U1*) that drives its enable signal. When the charge accumulated in the storage unit is above a fixed threshold (HTH, expressed in Volts), the enable signal rises, and the LDO starts feeding the load. The enable signal is kept high until the charge drops below the lower fixed threshold (LTH). This configuration allows the output conditioning unit to be decoupled during the recharge phase. The two thresholds are selected by means of a voltage divider corresponding to three resistor elements in the schematics: *R4*, *R5*, and *R6*. Those three resistance value can be adjusted according to the target sensing device.

### 3.2. Harvester Characterization

A common approach to increase the power harnessed using thermo-electric generator devices is to take several TEGs, and place them thermally and electrically in series, where each TEG represents a *stage*. In this way, each of the stages is exposed to a fraction of the total thermal gradient, therefore it generates less electrical power. However, the combination of the stages results in a greater total output power. We compared several configurations ranging from one up to five stages (results are not reported for the sake of brevity) to find the best trade-off between maximum output power and compactness. The time required to recharge an empty storage capacitor up to *HTH* with the two-stage configuration is 2.6× the time required by the three-stage solution. On the other hand, using the five-stage harvester, the recharge time is 1.5× faster with respect to the three-stage. We found that the *three-stage* setup realizes the best compromise for the specific target application. In the rest of this work, we present results obtained with this configuration.

**Figure 3:**
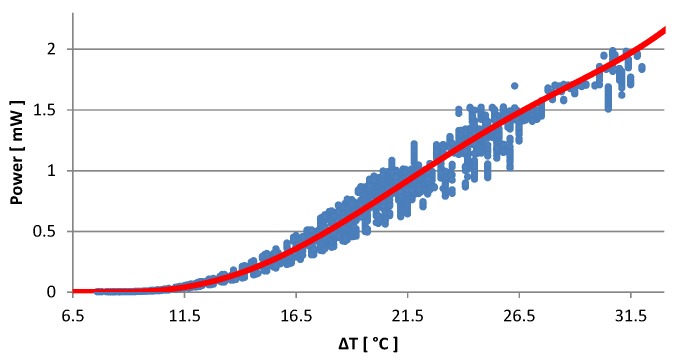
Generated output power (in Watts) *versus* Thermal gradient (in Celsius degrees).

**Figure 4:**
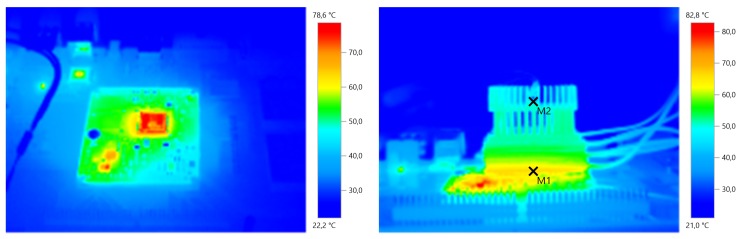
Thermal picture of the host equipped with the harvesting system during the experimental characterization. Left: seen from above; Right: side view.

The characterization has been performed directly on top of our target CPU, that is a 1.7 GHz dual-core ARM Cortex A15 mounted on an Arndaleboard. We measured the power generated for the whole range of working temperatures of the processor by varying the *host* CPU workload. Figure 3 shows the measured values of the generated output power in relation to the thermal gradient between the CPU package and the heat sink of the harvester. These results were used to extract, by cubic interpolation, the characteristic curve (in red) depicted on top of the data set (blue dots). The temperature of the ARM CPU has been measured by the *host* itself using its integrated temperature sensor, while the temperature on the heat sink is monitored by the wireless sensor node reading a thermistor placed in between heat sink fins. We verified the validity of those readings using a calibrated thermal imaging camera. An example of the temperature picture is reported in Figure 4. These experiments allowed us to confirm the thermal stability of the system. The results show that the harvesting module does not affect the thermal stability of the ARM CPU; conversely, the module allows the host to keep the maximum working temperature below the warning limit of 82 ${}^{{}^{\circ}}$C (the threshold used by the DVFS module of the Linux Kernel to scale down the working frequency) even with 100% load at its maximum clock speed (1.7 GHz). More details are given in Section 5.

To control the host CPU workload, we let the host processor run benchmark applications with different combinations of the clock frequency $f_{CLK}$, the percentage of activity dedicated to the task *CPU_load_*, and the duration $I_{task}$. The parameter ${CPU}_{load}$ considers the user CPU usage without the operating system overhead. From the measured data, we extracted the mathematical formula (see Figure 3 above) that models the input-output relation of the harvester. Moreover, we obtained the charge-discharge model of the storage unit, and the circuit efficiency (*η*), and we studied the relation between tasks (expressed as a combination of $f_{CLK}$, ${CPU}_{load}$, $I_{task}$) and harvested power.

To characterize the harvester, we focus our considerations on ${CPU}_{load}=\{0,25,50,75,100\}\%$, and $f_{CLK}=\;\{1.2,1.3,1.4,1.5,1.6\}$ GHz. Whatever the clock frequency, with no task in execution (meaning the CPU is Idle, ${CPU}_{load}=0\%$), the thermal gradient stabilizes around 8 ${}^{{}^{\circ}}$C. When ${CPU}_{load}$ is 25% or larger, the thermal gradient ΔT grows with the clock frequency. Also the task duration influences the amount of energy the system can generate. Generally, the transients of the CPU temperature last less than 120 s, but they are faster with higher clock frequencies. In fact, short tasks duration cannot increase the temperature as much as needed in order to observe a noticeable amount of power at the harvester output. For a CPU load ${CPU}_{load}=\left[25\%,50\%,75\%\right]$, whatever the CPU speed, a task that runs for more than two min generates around 3${\;}^{{}^{\circ}}$C greater thermal gradients with respect to short computational bursts. Under the same conditions, for a task with ${CPU}_{load}=100\%$, the thermal gradient may increase as much as 5 ${}^{{}^{\circ}}$C. When the CPU clock is set at its maximum speed, we observe the maximum value for ΔT reaching 30.31 ${}^{{}^{\circ}}$C, that matches with the maximum generated electrical power of about 2 mW (as shown in Figure 3). These values represent the boundary conditions that we use in the rest of the paper to evaluate the performance of the platform.

## 4. Case Study

A monitoring system for data centers is used to measure several environmental parameters whose value, when out of the ordinary, could denote that a hazard may be incurred. Different types of sensors, such as light or proximity detectors, can reveal the presence of unauthorized personnel. Moreover, by controlling the temperature value it is possible to infer whether there is an overheating problem, due for example to a fault in the cooling system. In our specific case, we aimed at the control of the ambient temperature, the heat sink temperature, the ambient light, the node orientation, and the state of charge of the supercapacitor that fed power to the monitoring device.

### 4.1. Prototype Design

**Figure 5:**
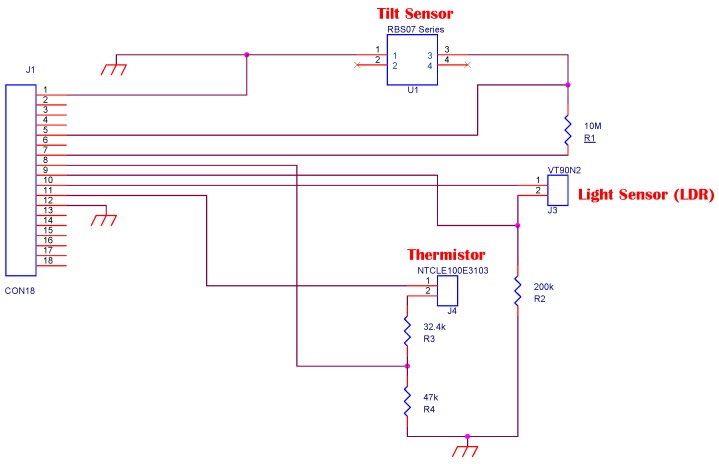
Schematic representation of the interconnection between the wireless node and the sensors.

Among the microcontroller based devices that lend themselves to this type of applications, that are fitted with radio, and show an energy budget in line with the availability provided from the TEG, we have identified the *TI eZ430-RF2500*. Several sensors have been connected to the microcontroller/ transceiver board according to the diagram reported in Figure 5. A Light-dependent Resistor (LDR), the *Excelitas Tech VT90N2* [24], has been used to measure light variations, and to spot the presence of personnel inside the server room. The LDR is connected to a voltage divider connected to the ADC mounted on the MSP430. To minimize the power consumption, the LDR circuit is powered only during measurement. The sensor stabilizes its value after only 76 ms. A tilt-sensor, the *DIP-RBS07 Series* [25], has been attached to the node, and it is used to detect if the server rack door has been opened. When the sensor stands on its correct position, the connection is closed. If it has been tilted, for example because the door has been opened, the sensor switches the electrical connection. A thermoresistor ([26]) is placed within the heat sink central fins to measure the temperature. The wireless node collects the environmental parameters, and sends the aggregated data to a central node. Moreover, the node can exploit the temperature information to infer the thermal gradient, and estimate the expected generated power of the TEG. Exploiting this prediction, the sensor node can determine autonomously at which rate to operate in order to stay alive, as described in Section 4.2. To reduce the power consumption, the monitoring node powers the sensors only when it is sampling their value.

### 4.2. Prototype Development

The *TI eZ430* board implements the proprietary *Simplicity* communication protocol stack, which provides advanced networking capabilities. We implemented a communication protocol that does not require setting up the radio link, the packet acknowledge, and token forwarding, since for the monitoring purposes a star-topology is most suitable, and allows the node to consume less energy when transmitting data.

The monitoring node is fed as soon as the voltage stored in the supercapacitor reaches HTH=3.08 V. The node boots with its registers settled for network configuration, timer usage and ADCs, and set to Low-Power Mode 3 (LPM3) with interrupts enabled. After a given amount of time, $I_{s}$, the microcontroller wakes up, and broadcasts a packet to a remote node (or to a PC) with the measured value for the heat sink temperature, light, orientation, and its voltage supply. Then in returns to *Idle* mode, and reiterates after $I_{s}$ seconds, assuming that the amount of energy available is enough to power it. When the voltage of the capacitor decreases under the threshold LTH=2.4 V, the comparator opens the path between the storage unit and the microcontroller, which consequently switches off.

We implemented two different policies to choose the value of $I_{s}$:

**Fixed time:**  in this implementation $I_{s}$ is fixed, and set equal to one second in our experiments. Therefore, if the supercapacitor contains enough power to supply the node, the node continuously transmits with a constant data rate;
**Temperature dependent:**  in the second implementation, the time between two successive transmissions is chosen by the sensor node according to the temperature measured on the heat sink of the harvester, which provides an indication of the amount of scavenged energy, used to estimate an optimal communication interval as described below.

In the first implementation, the node goes to deep low power mode in the time interval between two successive transmitting events, limiting the total power consumption. However, this implementation makes it possible for the node to drain enough power to bring the voltage on the storage unit below the LTH threshold, in case the harnessed energy is not enough. If this occurs, the node cannot operate until the available power returns larger than HTH.

**Figure 6:**
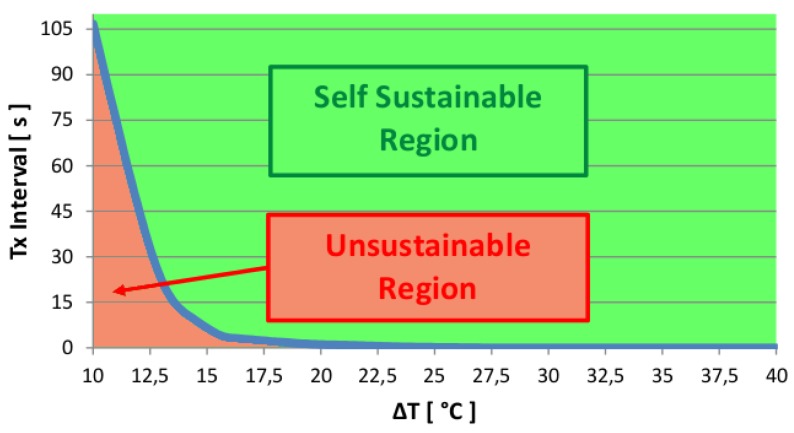
The Self-Sustainability curve represents the union of the points that separates the region of the plot where the nodes can operate autonomously from the region in which it fails to operate.

In the second implementation, the value of $I_{s}$ is a function of the heat sink temperature. Since the temperature on the heat sink is a function of the TEG system temperature, we can infer from it the amount of power the harvester is generating, and then predict the expected amount of charge that can supply the node. The node estimates the amount of power available through a look-up-table containing the data that express the relationship between temperature on the heat sink, and generated voltage, which was derived from characterization data extracted as described in Section 3.2. To evaluate the range of values within which the node can be self-powered, we compute the self-sustainability curve. The curve in Figure 6 represents the maximum transmission interval time that is feasible for thermal gradients in the range 10 °C–40 ${}^{{}^{\circ}}$C.

The region above the curve represents the self-sustainable region (S-S.R.), and corresponds to the condition in which the node can operate continuously, while the region below the curve corresponds to the unsustainable region (U.R.) and represents the transmission rates the node cannot operate at for longer periods of time. Knowing this data, it is possible to identify the optimal communication interval depending on the heat generated by the thermal source. For example, with 10 ${}^{{}^{\circ}}$C thermal gradient the shorter transmission interval is approximately 110 s, while at 15 ${}^{{}^{\circ}}$C it is possible to set a transmission rate lower than 10 s.

Tasks running on the host processor have been described according to three parameters: the clock frequency $f_{CLK}$, the percentage *CPU*_*load*_ of CPU time dedicated to the task, and the duration *I*_*task*_ of the process. This allows us to model every software as a combination of these three values, and to associate the corresponding CPU temperature. With $f_{CLK}$ = 1.2 GHz the mean heat sink temperature stabilizes around 31.8 °C, while the average CPU temperature is near 45 °C. The node transmits every 30 s even if it is possible to send every 13 s with a Δ *T* = 14 °C. As a consequence the system consumes less power than available, and the storage voltage increases. With the adjustable transmission rate implementation, after a certain period of time, the voltage in the storage capacitors tends to stabilize around 4.6 V, therefore the system is self-sustainable.

**Figure 7:**
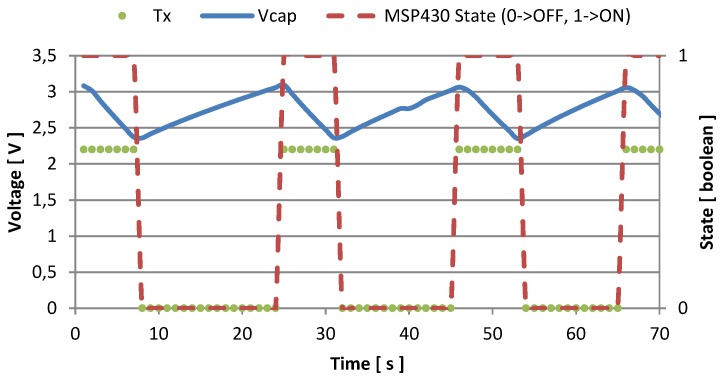
Voltage in the storage capacitance and transmission events with $f_{CLK}$ = 1.2 GHz, and ${CPU}_{load}=100\%$.

**Figure 8:**
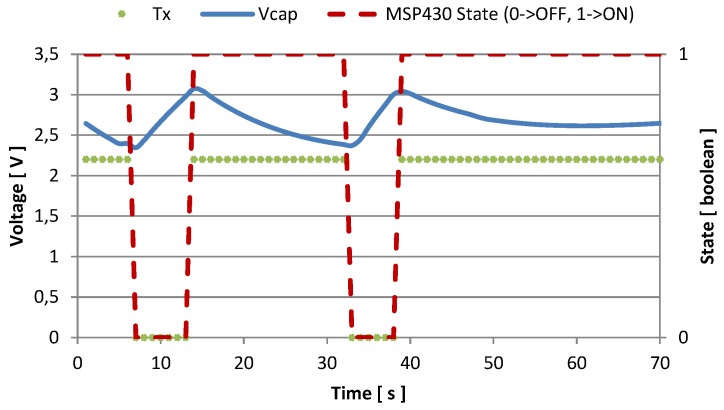
Voltage in the storage capacitance and transmission events with $f_{CLK}$ = 1.7 GHz, and ${CPU}_{load}=100\%$.

Every time the supply voltage crosses the high threshold, the node activates, and starts sampling and sending. In Figure 7 and Figure 8 we represented the amount of time the node is active, and the number of transmissions it is able to perform with two different values of $f_{CLK}$. When the host processor runs with $f_{CLK}$ = 1.2 GHz and *CPU*_*load*_ = 100%, the recharge time required to reach the voltage threshold is about 25 s, and after being activated, the monitoring unit is able to send data for 7 or 8 times before discharging the supercapacitor, and becoming inactive. This condition is represented in Figure 7, while Figure 8 shows measured data when the host processor clocks at its maximum speed: $f_{CLK}$ = 1.7 GHz and *CPU*_*load*_ = 100%. In the latter case, it is possible to perform continuous transmissions (every second) without discharging the supercapacitor once the temperature reaches its steady state. Moreover, just 6 s are required to recharge the storage unit.

**Figure 9:**
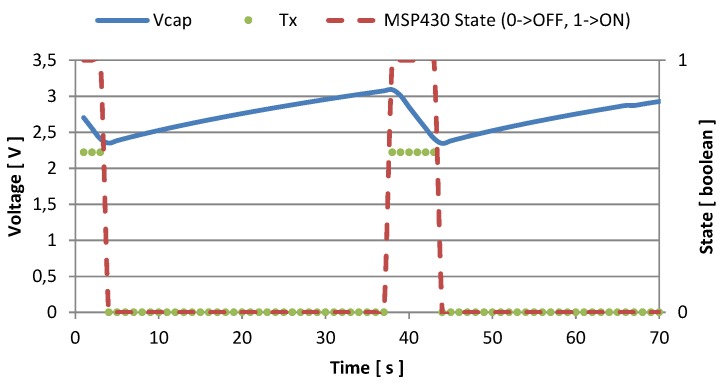
Voltage in the storage capacitance and transmission events with $f_{CLK}$ = 1.2 GHz, and ${CPU}_{load}=50\%$.

**Figure 10:**
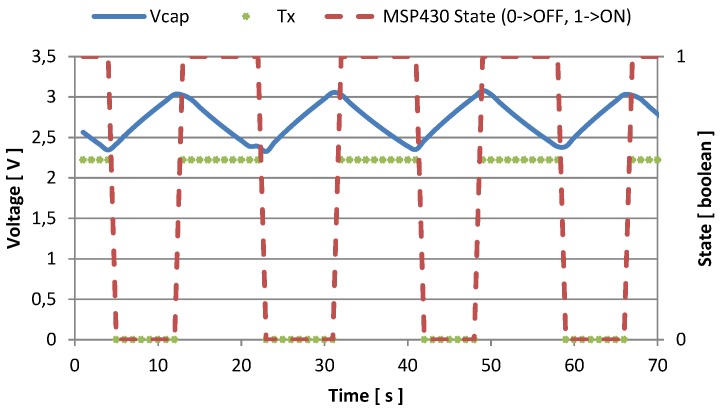
Voltage in the storage capacitance and transmission events with $f_{CLK}$ = 1.7 GHz, and ${CPU}_{load}=50\%$.

In Figure 9 and Figure 10 we show the activity of the node when the ${CPU}_{load}=50\%$ for $f_{CLK}$ = 1.2 GHz, and for $f_{CLK}$ = 1.7 GHz respectively, together with the voltage across the storage supercapacitor. Even with half the CPU load, the energy harvesting system is able to recharge the storage supercapacitor. For $f_{CLK}$ = 1.2 GHz, the monitoring system can sample the sensors and send the data six times before going under threshold. After that, 33 s are required to restore the charge. For $f_{CLK}$ = 1.7 GHz, the monitoring unit senses and sends eleven times and switches off for 8 or 9 s before resuming.

**Figure 11:**
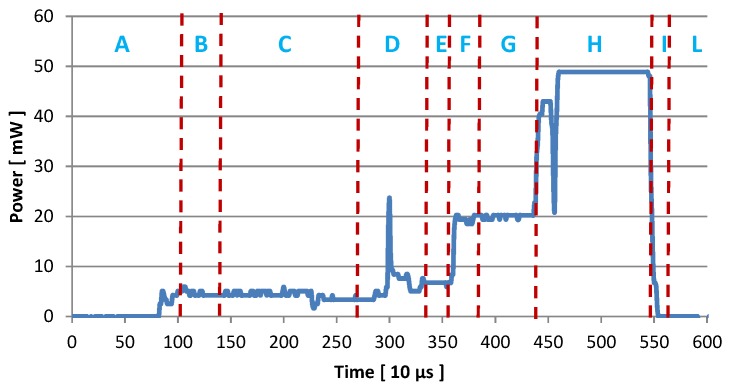
Measured power consumption of the prototype node during a sense and send event.

The energy spent by the node has been characterized by measuring the voltage across a resistor $R_{meas}$ placed in series between the sensing node and the power supply circuitry. Through this approach, and by knowing the resistance $R_{meas}=10.2\;\Omega$, we computed the effective power consumption. By analyzing how the values of the power consumption evolve with time, we also associated the specific task the node is performing to the corresponding amount of energy spent. Table 1 summarizes the task specific power consumption value as extracted from Figure 11. From Table 1 it is possible to compute the amount of energy required by the system to perform a single sense-and-send operation, that is $E_{TX}=84.69\;\mu$J. Moreover, knowing that for each send operation the radio is used for $I_{TX}=4.86$ ms, the total current consumption of the *MSP430* board in *Idle* mode together with its radio module *CC2500* is [27,28]: $MSP430_{IDLE}+CC2500_{IDLE}=1.3\;\mu$A. The power consumption is: $P_{IDLE}=2.2$ V × 1.3 μA =2.86μW. To supply the system in *Sleep* mode for one second 2.84μJ are needed. Summing all together, to run the application on the board for one second, $E_{tot}=87.53\;\mu$J is the required total energy.

**Table 1:** Power consumption of the *eZ430-RF2500* board during a sense-and-send event.

Symbol	Event	$\bar{I}$ mA	Time ms	P mW	E μJ
A-L	Idle	$1.3\times 10^{-3}$	-	-	-
B	Temp & Vcc	1.94	0.38	4.26	1.61
C	NTC Temp	1.94	1.37	4.26	5.83
D	Calc & XOSC Startup	2.56	0.70	5.63	3.94
E	Ripple Counter	3.02	0.21	6.64	1.39
F-G	Msg & PLL calib.	8.29	0.87	18.23	15.86
H	Tx Mode	20.95	1.19	46.09	54.84
I	Switch LPM	3.95	0.14	8.69	1.21

## 5. Results

**Figure 12:**
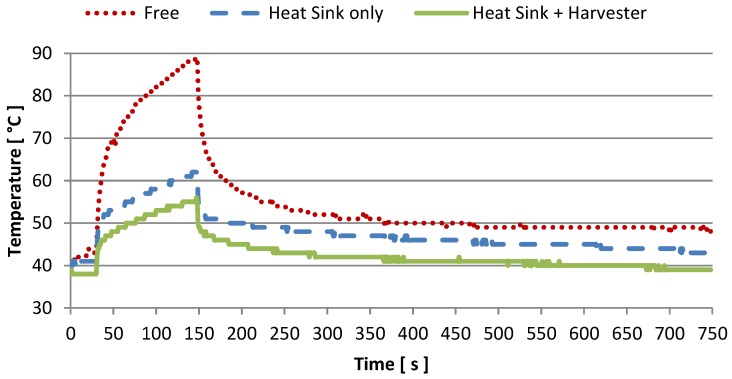
CPU temperature trend in three configuration: with and without the heat sink, and with the proposed prototype on top.

To ensure that the proposed systems does not adversely affect the reliability of the server, we evaluated how the harvester and the sensor node influence the thermal dissipation characteristic of the target host CPU. In our experiment, we launched a process with ($f_{CLK}$, *CPU_load_*, *I_task_*) = (1.7 GHz, 100%, 120 s) and measured the CPU temperature in three scenarios: the CPU without passive dissipation (as sold by the vendor), with the heat sink on top (the same heat sink mounted on top of the harvesting module), and with the complete proposed prototype. The experiment is used to understand whether the system causes the CPU to overheat. On the contrary, it turns out that our prototype allows the CPU to work at lower, safer temperatures. Results are depicted in Figure 12. As the reader can easily notice, the temperature of the CPU without heat sink rapidly reaches a dangerous value (about 90 °C). The presence of the heat sink allows the temperature to not exceed 62 °C. Even better, when the complete harvester is mounted the temperature reaches only 55 °C. Therefore, the proposed system is an excellent passive heat sink which ensures a reduction of temperature of about 5 °C in Idle mode, and up to 7 °C when the target host runs at its maximum performance. Compared to the host deprived of the heat sink, the harvester guarantees to cool the CPU by about 35 degrees. This is contrary to other similar applications where the presence of a TEG in between a desktop CPU package and the heat sink raises the CPU temperature by about 10 °C–20 °C [14,15].

The application with fixed sampling period $I_{s}=1$ s is self-sustainable when the thermal gradient is larger than ΔT=12${}^{{}^{\circ}}$C, corresponding to a CPU temperature of about 35 ${}^{{}^{\circ}}$C. The dynamic (or adaptive) $I_{s}$ implementation preserves energy when the temperatures are low, and allows the system to increase the sampling rate when temperatures increase. For thermal gradients smaller than 12.5 ${}^{{}^{\circ}}$C, the system works with sampling period $I_{s}=109$ s, while with 15 ${}^{{}^{\circ}}$C the period decreases to around 6 s, which is reasonable for the target application of the WSN. When ΔT≥22.5 ${}^{{}^{\circ}}$C, the time interval is set to $I_{s}=1.27$ s, therefore the data rate is comparable with the fixed sampling period implementation. For thermal gradients above 25 ${}^{{}^{\circ}}$C the system is self-sustainable with a transmission interval as small as $I_{s}=0.35$ s.

**Figure 13:**
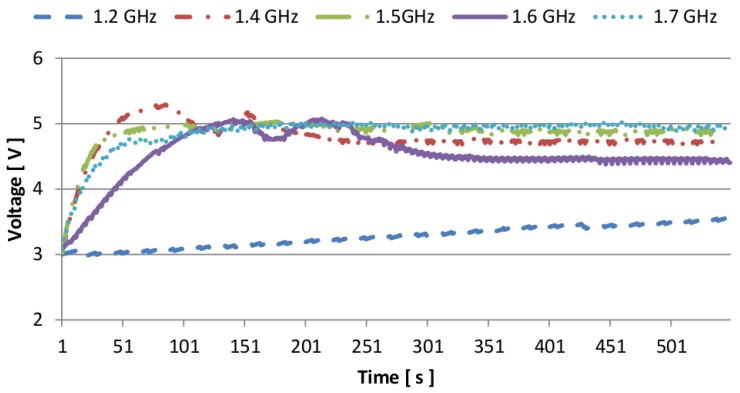
Measured supercapacitor voltage using adaptive sampling rate for different clock frequencies of the host processor.

The dynamic (or adaptive) application has been tested to evaluate its effectiveness under different working conditions. Results are summarized in Figure 13, in which we focused on the trend of the capacitor charge upon reaching the threshold of operation of the node (*i.e.*, HTH). The plot shows that the implementation requires some initial time to reach a steady state. During this time, of a few minutes, the heat has not propagated uniformly, therefore the temperature readings are not accurate and the voltage inside the storage unit tends to fluctuate, but never decreases below the HTH threshold. After the temperature stabilizes, the system is always switched on and the voltage across the supercapacitor increases continuously toward the maximum value of 5 V. Interestingly, for $f_{CLK}\;=\;1.6$ GHz the curve does not follow the increasing trend, and instead stabilizes around 4.5 V. This happens because the resulting thermal gradient ΔT is mid way between two values in the look-up-table the microprocessor uses to choose the sampling rate $I_{s}$, which are selected alternatively. As a consequence, the voltage tends to fluctuate and never reaches 5 V, since in this case the node consumes more power than what the harvester generates.

The storage capacitance must be sized according to the application the node has been programmed to perform, the efficiency of the harvester, the thermal characteristics of the *host* processor, and the policy of data collection rate. In fact, in the prototype device described in previous sections, by using a storage supercapacitor SC1=1 F, 67 min are required to fully charge the empty storage unit with the *host* configured with $f_{CLK}\;=\;1.7$ GHz and ${CPU}_{load}=100\%$. When the capacitor is full the node is able to perform 12 thousand transmissions (with one second interval) after the *host* is switched off. With the same conditions, the prototype equipped with SC1=2.2 mF recharges in 53 s, but is able to transmit only five times after the *host* has been switched off.

While the basic networking infrastructure is in place, in this paper we have focused primarily on experiments involving a single server and corresponding sensor and heat sink. A realistic implementation in a data center requires the deployment of several sensors nodes, resulting in possible contention of the network resources. Thus, we expect that an increase in the complexity of the wireless communication protocol, including for example clear channel assessment and acknowledges to deal with larger WSNs deployments, would results in a performance loss, causing a higher power consumption of the nodes. At the same time, in order to support a realistic implementation, we expect to increase the energy recovery efficiency by pushing forward the components integration and by relying on an automatic manufacturing process. Both directions need further research and investigation before the system can effectively be deployed in a real data center environment. Nonetheless, this prototype marks a significant step forward in terms of efficiency, autonomy and size with respect to the state of the art.

## 6. Conclusions

We presented a self-powered WSN node for data center monitoring. We described the design of the device, starting from the selection of the proper TEG that fits over the ARM CPU package, including the design and development of an electronic circuit and PCB for the conditioning, storage and management of power, and the adoption of a radio-equipped microprocessor device. We discussed the methodology and the results of the prototype characterization that has been conducted considering as a target device a high performance ARM board chosen as representative for the thermal characteristic of the future low-power data centers architecture. The prototype has been programmed to perform the application in two flavors: a fixed interval implementation, and a dynamic data rate that adjusts with the available energy. Results are promising and demonstrate that the proposed system is able to self-power itself when placed in the environment it has been designed to monitor, providing distributed tracking of a large infrastructure without affecting the power budget of the facility. The system, and the application, can be adapted to the target host architecture by adopting the design methodology described in this work. The sensing node can be extended by adding other functionalities at the cost of a larger power requirement. With our approach it is feasible to integrate other kind of sensors (such as power meters, low-power imagers, or volatile pollution monitors) without impacting system reliability by properly tuning the circuit parameters.
